# Consistency of spatial ability performance in children, adolescents, and young adults

**DOI:** 10.3389/fpsyg.2024.1365941

**Published:** 2024-02-28

**Authors:** Christina Morawietz, Nils Dumalski, Anna Maria Wissmann, Jonas Wecking, Thomas Muehlbauer

**Affiliations:** Division of Movement and Training Sciences/Biomechanics of Sport, University of Duisburg-Essen, Essen, Germany

**Keywords:** visual-spatial abilities, reliability, reproducibility, practical relevance, youth

## Abstract

**Background:**

Spatial abilities are essential cognitive skills for many aspects of our everyday life that develop substantially throughout childhood and adolescence. While there are numerous measurement tools to evaluate these abilities, many of them have been designed for specific age groups hampering comparability throughout development. Thus, we determined test–retest-reliability and minimal detectable change for a set of tests that evaluate spatial ability performance in their variety in youth and compared them to young adults.

**Methods:**

Children (age: 11.4 ± 0.5 years, *n* = 26), adolescents (age: 12.5 ± 0.7 years, *n* = 22), and young adults (age: 26.1 ± 4.0 years, *n* = 26) performed a set of five spatial ability tests twice, 20 min apart: Paper Folding Test (PFT), Mental Rotation Test (MRT), Water Level Task (WLT), Corsi Block Test (CBT), and Numbered Cones Run (NCR). Relative and absolute test–retest reliability was determined by calculating the intraclass correlation coefficient (ICC_3,1_) and the standard error of measurement (SEM), respectively. Further, the minimal detectable change (MDC_95%_) was calculated to identify clinically relevant changes between repeated measurements.

**Results:**

Irrespective of test, reliability was “excellent” (i.e., ICC_3,1_ ≥ 0.75) in all age cohorts and the SEM values were rather small. The MDC_95%_ values needed to identify relevant changes in repeated measurements of spatial ability performance ranged between 0.8 and 13.9% in children, 1.1 and 24.5% in adolescents, and 0.7 and 20.8% in young adults.

**Conclusion:**

The determined values indicate that the investigated set of tests is reliable to detect spatial ability performance in healthy children, adolescents, and young adults.

## 1 Introduction

Gaining independence and learning to lead an autonomous life are key aspects when growing up. Spatial abilities play an essential role in this process and are encountered frequently in our everyday life (Claessen et al., 2016; Fernandez-Baizan et al., 2019). They are involved when we need to find a way to a distant destination, when we have to orientate ourselves in unknown environments or when we need to remember where we left our keys (Tzuriel and Egozi, 2010; Fernandez-Baizan et al., 2019). Spatial abilities are considered primary cognitive abilities, that start to develop from early childhood onwards and reach an adult-like level in adolescence (Fernandez-Baizan et al., 2019; Newcombe, 2019). While growing up, age-appropriate spatial abilities are an indicator for a healthy developing brain (Leplow et al., 2003). In addition to that, good spatial abilities have been associated with higher academic achievements, particularly in STEM-Subjects (science, technology, engineering, mathematics) (National Research Council, 2006; Newcombe, 2010; Ishikawa and Newcombe, 2021). As spatial abilities can be enhanced by training, it should be of utmost importance to evaluate and facilitate these skills on a regular basis in children and adolescents (Uttal et al., 2013).

However, the concept of spatial ability is interpreted in many different ways by the scientific community (Voyer et al., 1995; Heil, 2020). While there is agreement, that spatial abilities can be subdivided into different components, the amount, definition, naming, and testing of these subfactors varies widely across researchers (D'Oliveira, 2004; Quaiser-Pohl et al., 2004; Yilmaz, 2009). A recent review by Uttal et al. (2024) extensively addresses several of these issues, like the current lack of reliable and valid spatial ability tests, the difficulty to access tests, the lack of tools that can be applied across age groups as well as the inconsistency in the research society as to which spatial construct each test is supposed to measure,. A well-known and frequently used categorization of spatial abilities is the one developed by Linn and Petersen (1985). They describe spatial abilities using the labels spatial perception, mental rotation, and spatial visualization. Here spatial perception describes the determination of spatial relationships with respect to the orientation of the own body. Distracting information might also be included. Mental rotation is defined as the ability to rotate rapidly and precisely two-or three-dimensional figures. Spatial visualization describes the complex, multistage processing of spatial information which might be solved with different approaches (Linn and Petersen, 1985). A more recent approach by Newcombe and Shipley (2015) distinguishes between static and dynamic and intrinsic and extrinsic spatial skills that can be combined in a 2 × 2 matrix (Uttal et al., 2013) resulting in the following categories: intrinsic-static (i.e., identifying spatial characteristics of an object), intrinsic-dynamic (i.e., modification of the spatial characteristics of an object like folding or rotation), extrinsic-static (i.e., identifying the spatial location of an object in relation to the environment), and extrinsic-dynamic (i.e., modification of the relation of objects to one another or the viewer due to movement). Linn and Petersen’s categorization is represented in this newer approach to some extend (Uttal et al., 2013).

Over the years, various measurement tools have been developed to evaluate the different spatial abilities. While some have originally been developed for children but are also used in adults, others have originally been developed for adults but have also been applied in younger populations (Vandenberg and Kuse, 1978; Merriwether and Liben, 1997; Hoyek et al., 2012). However, psychometric data on the use of spatial tests in different age groups are scarce (Uttal et al., 2024). Due to the lack (or lack of availability) of suitable, age appropriate and comparable measurement tools, numerous adaptations and adjustments have been developed for many of these outcome measures (e.g., use of pictures instead of multidimensional figures, more or less options to answer from, etc.) by individual researchers (Hoyek et al., 2012; Jansen et al., 2013; Uttal et al., 2024). As a result, there is no consistency in the tools used to evaluate spatial abilities of children and adolescents in the current literature. This severely impedes the evaluation of spatial abilities throughout the developmental process as well as the comparability of spatial abilities between age groups.

The measurement tools most frequently used in spatial ability research are easy to administer and well-replicable paper-and-pencil tests. However, most of these paper-and-pencil-tests (e.g., Mental Rotation Test [MRT], Paper Folding Test [PFT]) only depict some aspects of spatial abilities, namely small-scale spatial abilities (i.e., the ability to mentally manipulate small figures or objects, commonly performed from a single viewpoint) (Hegarty et al., 2006; Heil, 2020). To get a more comprehensive view on the spatial abilities of a person, more factors should be taken into consideration. Large-scale spatial abilities (i.e., the ability to process spatial information of the real environment including perspective changes of the viewer) (Hegarty et al., 2006; Yuan et al., 2019) have been less researched for many years (Quaiser-Pohl et al., 2004; Hegarty et al., 2006). Most frequently they are assessed using real world navigation tasks, learning new environments (real world or map), estimating directions, or using virtual environments (Schmelter et al., 2009; Wang et al., 2014; Heil, 2020). Consequently, assessments are customized to the respective environments or require large spaces making it difficult to control and standardize the conditions for these kinds of tests (Uttal et al., 2024). This hampers their reproducibility, comparability, and feasibility for many researchers (Jansen-Osmann, 2007; Schmelter et al., 2009). The more recent development of VR-based environments for navigational assessments could be a solution to this problem, however to date only few of these assessment tools exist and even less have open access (Uttal et al., 2024). Moreover, VR-equipment is not available to all researchers. Still, for a complete impression of spatial abilities the assessment of skills on a larger scale should not be left out of consideration. Moreover, visuospatial working memory should be taken into account, as it appears to be closely linked to real world orientation ability (Coluccia, 2008; Nori et al., 2009; Mitolo et al., 2015).

It further needs to be taken into consideration that spatial ability tests need to be selected carefully, when conducting spatial ability research. Some tests that claim to evaluate different spatial constructs in fact measure the same skills, while other tests evaluate very different constructs even though their names sound alike (Hegarty and Waller, 2005; Uttal et al., 2024). This makes it difficult to make the right choices when selecting spatial ability tests for a research project (Uttal et al., 2024). It should also be acknowledged that a variety of aspects of spatial skills cannot be tested with currently available spatial tests (Newcombe and Shipley, 2015; Uttal et al., 2024).

Regarding the difficulties for spatial testing discussed above, the aim of this study is to determine the test–retest-reliability and minimal detectable change of a set of established tests that evaluate spatial abilities in their variety. To investigate the role of age and suitability for different age groups, tests will be performed with healthy children and adolescents and compared to young adults. The choice of tests was guided by different parameters, e.g., tests had to be well established and researched before, tests had to be accessible and tests had to be easy to administer within the study setting (school and university). As it is still used frequently in research, our choice of tests was built upon the framework by Linn and Petersen (1985) and extended by a real-world orientation test that might be allocated to the extrinsic-dynamic category of Newcombe and Shipley (2015). Moreover, we integrated a test of visuospatial working memory. We expected that the results of spatial ability testing would be reproducible in youth and young adults.

## 2 Methods

### 2.1 Participants

A total of 74 healthy subjects volunteered to participate in this study. Children (*n* = 26, 13 females, 13 males, age: 11.4 ± 0.5 years) and adolescents (*n* = 22, 13 females, 9 males, age: 12.5 ± 0.7 years) attended public secondary schools in the Ruhr area of North Rhine-Westphalia, Germany while the young adults (*n* = 26, 11 females, 15 males, age: 26.1 ± 4.0 years) were recruited from the University of Duisburg-Essen, Essen, Germany. An *a priori* power analysis with G*Power, version 3.1.9.7 (Faul et al., 2007) showed that a total of 67 participants would be required. The analysis was run with *ρ* = 0.30, *α* = 0.05, 1-*β* = 0.80. None of the participants had previous experience with the performed set of tests. Prior to the start of the investigation, written informed consent was obtained from all participants and their legal guardians if required. The study was conducted with approval (EA-PSY20/23/04102023) of the Human Ethics Committee of the University of Duisburg-Essen, Faculty of Educational Sciences.

### 2.2 Procedures

**Figure 1:**
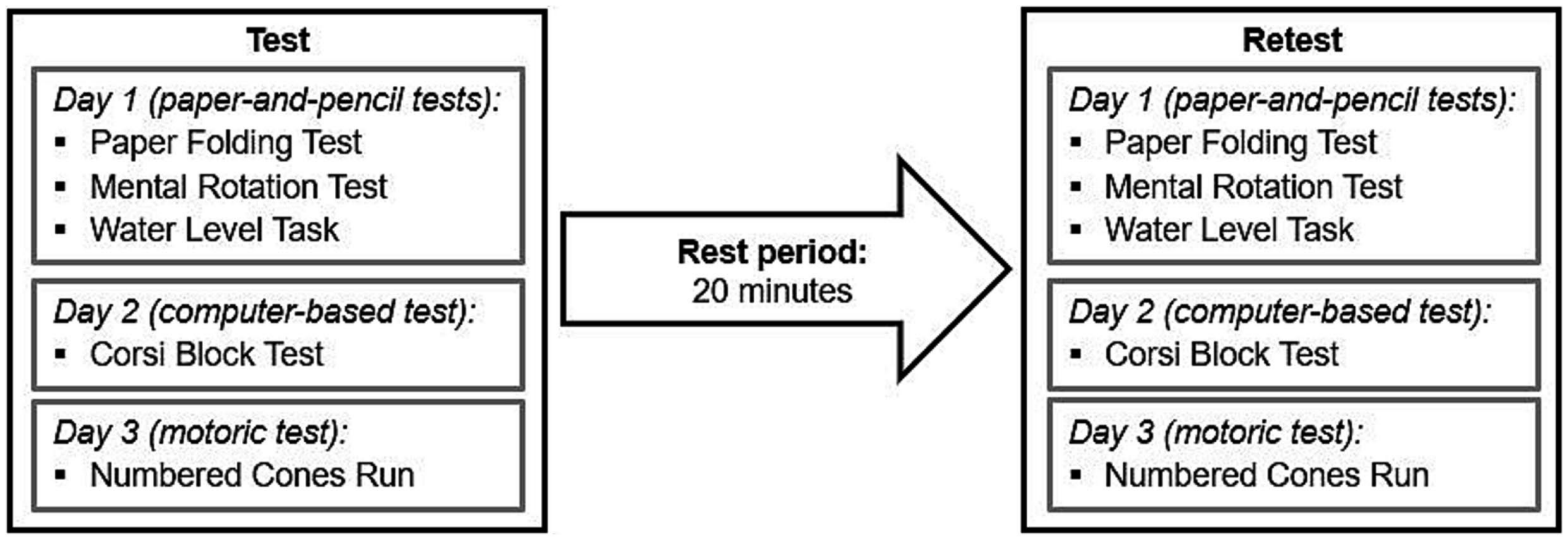
Schematic diagram of the experimental procedure.

All participants completed a set of five spatial ability tests. Data were collected within the school-or university-setting and took place during regular class times. The procedures were spread over three testing days (Figure 1). Prior to each test, the participants received standardized verbal instructions. On the first day, three paper-and-pencil tests were performed within the classroom setting. Each participant received a test booklet and performed the tests individually. On the second day, participants performed a computer-based test. They were instructed in small groups of a maximum of five students. The test was then conducted in a one-on-one situation in a separate quiet room within the school or university. On the third day, a motoric test was conducted in small groups of five students in the gym of the school or university. All tests were repeated after a 20-min break.

### 2.3 Measures

#### 2.3.1 Paper Folding Test (PFT)

The PFT (Ekstrom et al., 1976) evaluates spatial visualization. Participants were asked to mentally follow the process of a sheet of paper being folded, a hole punched through it and the paper being unfolded again. They saw a picture of a sheet of paper which was folded and a picture of the same paper with a hole punched through it. Participants then had to decide which out of five options showed the correct unfolded piece of paper. The test consisted of two parts with ten items each and had a time-limit of three minutes for each part with three minutes break in between. A visual demonstration with a model paper was performed during instructions. The test included one practice item that was to be completed prior to the first part. Every correct answer was scored with one point, resulting in a maximum of 20 points.

#### 2.3.2 Mental Rotation Test (MRT)

The MRT (Vandenberg and Kuse, 1978) examines the ability to mentally manipulate three-dimensional block figures. This includes the mental rotation, mirroring, and tilting of these objects. Version A by Peters et al. (1995b) was used. Participants saw a picture of a target object on the left and were asked to decide which two out of four sample objects were rotated versions of the target object. The test was composed of two parts with twelve items each. There was a time-limit of three minutes for each part with a three-minute break in between. During instructions, a visual demonstration of the task (i.e., rotation, tilting, mirroring) was performed with a block figure built out of small cubes. Moreover, four practice items were performed prior to the test (time-limit: five minutes). Participants received one point if both answers per task were correct. A maximum of 24 points could be achieved in total.

#### 2.3.3 Water Level Task (WLT)

The WLT (Piaget et al., 1956) is a tool to determine spatial perception. It was designed to examine the development of spatial abilities in children. As the test has only been described anecdotally, the version by Yingying Yang (University of Alabama) (Merrill et al., 2016) was used in this study. Participants saw a jar half-full of water and twelve empty jars at different levels of inclination. They were asked to imagine that each of the empty jars is half-full of water and then draw a line representing the water level in the jar. Participants saw an illustration of an empty jar that could be tilted in different inclinations for clarification during instructions. The time-limit for this task was three minutes. Participants received one point if the line they drew was within the tolerance range of ±10° from horizontal. A maximum of 12 points could be scored.

#### 2.3.4 Corsi Block Test (CBT)

The CBT (Corsi, 1972) captures the visuospatial short-term-and working memory as well as spatial learning. A computer-based and self-programmed version of the test was used based on the online-demo of Millisecond Software, Seattle, USA. The positioning of the blocks and the sequences tested were in accordance with Kessels et al. (2000). Participants saw a black screen with nine blue squares on it. In predefined sequences some of the blue squares then lightened up in yellow. The sequences had to be repeated immediately at the click of a mouse. The first two sequences comprised of two squares. For every following level, one square was added to the block sequence until a span of nine squares was reached. Participants had two trials per block sequence of the same length. At least one sequence per level had to be repeated correctly in order to reach the next level. If both block sequences of the same length were not repeated correctly, the test ended immediately. During instructions, participants saw an illustration of the testing screen for clarification. There was no time-limit. The block span (i.e., length of the last correctly reproduced block sequence with a maximum of 9 points) as well as the total score (i.e., maximal block span X amount of correctly reproduced sequences with maximum 144 points) were recorded and used for further analyses.

#### 2.3.5 Numbered Cones Run (NCR)

**Figure 2:**
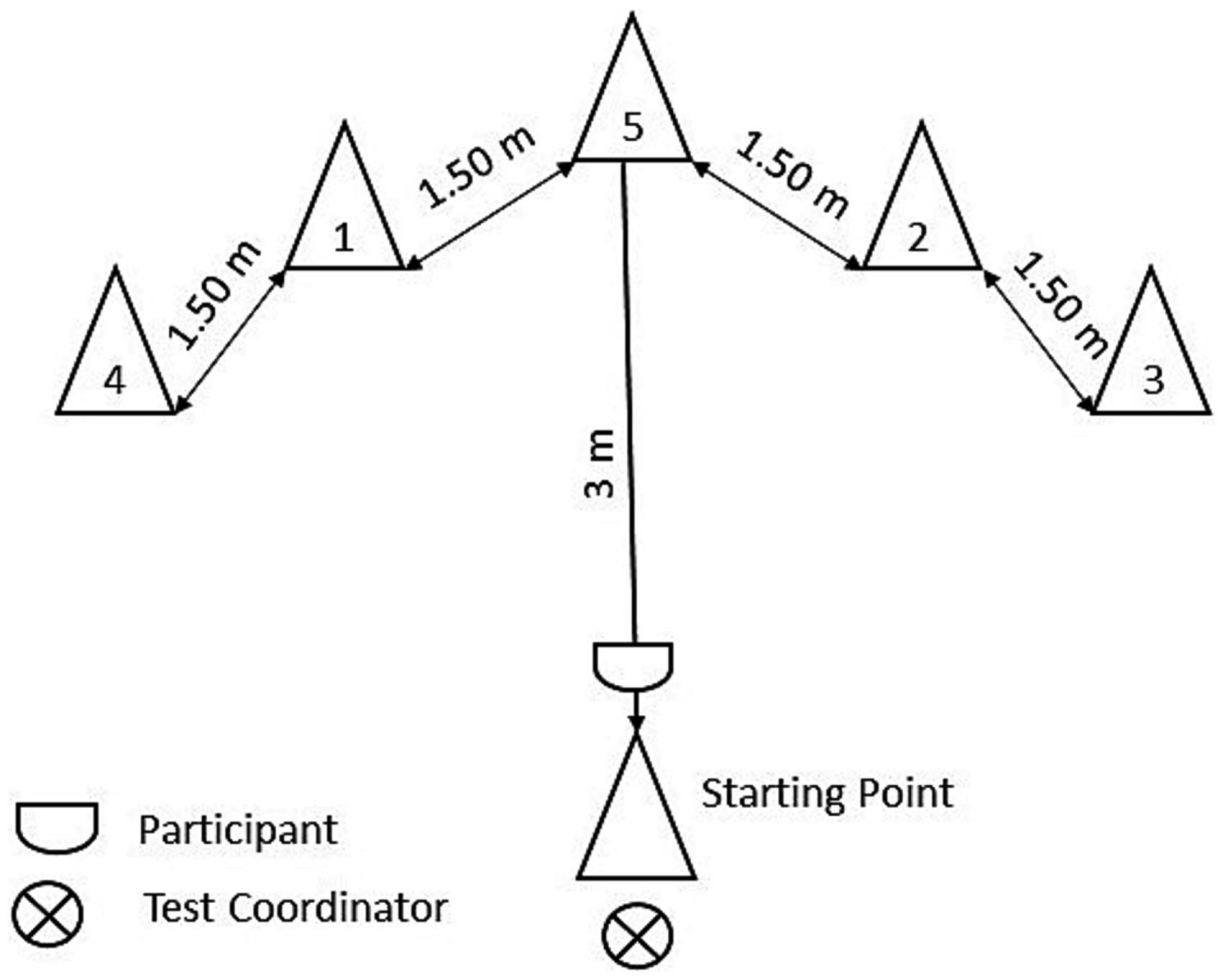
Schematic depiction of the Numbered Cones Run.

The NCR, an adaptation of the Medicine Ball Number Run (Jung, 1983 as cited in Hirtz et al., 2010) evaluates spatial orientation. Five numbered cones were placed in random order in a semicircle 1.5-m apart from each other. Another cone marked the starting point at 3-m distance (Figure 2). Participants stood at the starting point facing away from the numbered cones. A number was then called out and participants were asked to run to the respective cone, touch it and return to the starting point. Right before the starting point was reached, a second number was called out. This process was repeated for a third time. Participants had two trials and the mean value of both trials was used for further analysis. The order of cone numbers differed for every participant and the three numbers called out were determined by a random number generator in advance. During instructions, participants saw an illustration of the set-up for clarification.

### 2.4 Statistical analysis

For all data, group mean values and standard deviations (*SD*) were calculated. The intraclass correlation coefficient (ICC_3,1_) and the 95% confidence interval (CI) were used to determine the relative reliability (i.e., the degree to which individuals maintain their position in a sample with repeated measurements) (Weir, 2005). In accordance with the classification of Fleiss (1999) ICC ≥ 0.75 was considered “excellent,” 0.40 ≤ ICC < 0.75 was considered “moderate-to-good,” and ICC < 0.40 was considered “poor.” The absolute reliability of the data (i.e., the degree to which repeated measurements vary for individuals) was assessed using the standard error of measurement (SEM) that estimates the amount of error related with the measurement (Weir, 2005). The minimal detectable change (MDC_95%_) was calculated to identify clinically relevant effects between repeated measurements of one subject (Weir, 2005; Haley and Fragala-Pinkham, 2006). All statistical analyses were performed with Statistical Package for Social Sciences version 27.0.

## 3 Results

Means and *SD*s for spatial ability performance during the test and retest assessment by age cohort are presented in Table 1. Table 2 illustrates the statistics for relative and absolute reliability of the data. Specifically, the ICC_3,1_ values ranged between 0.78–0.95 in children, 0.76–0.94 in adolescents, and 0.78–0.90 in young adults, which is indicative of “excellent” relative test–retest reliability. Additionally, the SEM values ranged from 0.3 to 5.0 in children, from 0.4 to 8.8 in adolescents, and from 0.3 to 7.5 in young adults. Lastly, Table 3 shows the MDC_95%_ values that ranged from 0.8 to 13.9% in children, from 1.1 to 24.5% in adolescents, and from 0.7 to 20.8% in young adults.

**Table 1:** Spatial ability performance for the test and retest assessment by age cohort.

Outcome	Children (*n* = 26)		Adolescents (*n* = 22)		Young adults (*n* = 26)	
	Test	Retest	Test	Retest	Test	Retest
Paper Folding Test [pt.]	7.0 ± 3.2	9.4 ± 3.4	8.6 ± 4.0	9.0 ± 4.7	10.2 ± 4.2	12.5 ± 3.8
Mental Rotation Test [pt.]	7.2 ± 3.5	9.7 ± 5.8	9.5 ± 4.6	9.3 ± 4.9	9.9 ± 4.7	12.3 ± 5.4
Water Level Task [pt.]	4.9 ± 3.4	6.4 ± 3.4	8.6 ± 3.0	9.4 ± 3.1	8.7 ± 2.9	9.5 ± 2.6
Corsi Block Test span [pt.]	5.4 ± 1.3	5.4 ± 1.2	5.5 ± 0.9	5.9 ± 1.1	6.4 ± 1.1	6.8 ± 1.1
Corsi Block Test CS [pt.]	42.2 ± 17.7	43.5 ± 19.6	45.4 ± 14.9	53.5 ± 20.2	63.2 ± 22.7	72.9 ± 23.4
Numbered Cones Run [s]	10.5 ± 1.2	9.8 ± 1.2	9.0 ± 1.2	8.3 ± 1.0	9.5 ± 0.9	9.4 ± 0.8

Values are means ± standard deviations. CS, composite score.

**Table 2:** Intraclass correlation coefficient with 95% confidence interval and standard error of measurement by age cohort.

Outcome	Children (*n* = 26)		Adolescents (*n* = 22)		Young adults (*n* = 26)	
	ICC_3,1_ (95% CI)	SEM	ICC_3,1_ (95% CI)	SEM	ICC_3,1_ (95% CI)	SEM
Paper Folding Test [pt.]	0.78 (0.51–0.90)	1.6	0.94 (0.86–0.98)	1.1	0.87 (0.71–0.94)	1.5
Mental Rotation Test [pt.]	0.81 (0.58–0.92)	2.1	0.84 (0.62–0.93)	1.9	0.84 (0.63–0.93)	2.1
Water Level Task [pt.]	0.88 (0.74–0.95)	1.2	0.87 (0.70–0.95)	1.1	0.78 (0.50–0.90)	1.3
Corsi Block Test span [pt.]	0.95 (0.88–0.98)	0.3	0.84 (0.65–0.93)	0.4	0.85 (0.65–0.93)	0.4
Corsi Block Test CS [pt.]	0.93 (0.84–0.97)	5.0	0.76 (0.42–0.90)	8.8	0.90 (0.77–0.95)	7.5
Numbered Cones Run [s]	0.91 (0.79–0.96)	0.4	0.86 (0.65–0.94)	0.4	0.90 (0.77–0.95)	0.3

CI, confidence interval; CS, composite score; ICC, intraclass correlation coefficient; SEM, standard error of measurement.

**Table 3:** Minimal detectable change by age cohort.

Outcome	Children (*n* = 26)	Adolescents (*n* = 22)	Young adults (*n* = 26)
Paper Folding Test [pt.]	4.5	2.9	4.1
Mental Rotation Test [pt.]	5.9	5.2	5.8
Water Level Task [pt.]	3.3	3.0	3.6
Corsi Block Test span [pt.]	0.8	1.1	1.2
Corsi Block Test CS [pt.]	13.9	24.5	20.8
Numbered Cones Run [s]	1.1	1.2	0.7

CS, composite score.

## 4 Discussion

In the present study, test–retest reliability of a set of five tests that evaluate spatial abilities in their variety was investigated in healthy children, adolescents, and young adults. As expected, and in parts in accordance with previous literature, the testing of spatial ability resulted in reproducible performances in youth and young adults.

Given that the availability of previous literature on test–retest reliability varies considerably between the five tests, results from different age groups or different statistical test–retest analyses like Pearson’s product moment correlation coefficient (PCC) need to be consulted for comparison. With reference to Taylor (1990) PCC values were interpreted as weak (*r* = 0.10 to 0.35), moderate (*r* = 0.36 to 0.67), or strong (*r* = 0.68 to 1.00). Therefore, strong PCC values will be considered comparable with excellent ICC values, moderate PCC values will be considered comparable with moderate-to-good ICC values, and weak PCC values will be considered comparable with poor ICC values.

### 4.1 Paper Folding Test

The PFT delivered excellent ICC values across all age groups (0.78–0.94) and rather small SEM values ranging from 1.1 to 1.6 points indicating a good precision of the test by specifying how far measurement errors spread around a true score that is estimated from the derived scores (Musselwhite Thompson and Wesolowski, 2018). Unfortunately, no appropriate study on test–retest reliability of this test in children or adolescents exists in the current literature. Therefore, other age groups were consulted as basis for discussion. In line with our findings, Salthouse and Tucker-Drob (2008) tested 227 adults (age range: 18–97 years) and report test–retest correlations of *r* = 0.77. They did however perform the retest according to the participants’ schedule (mean: 6.7 days) and extend the time limit to ten minutes (Salthouse et al., 2006) which might have impacted on their results as participants had more time for recovery between tests. The extended time frame allowed participants to attempt to solve more items and revise each item even more thoroughly. Even higher PCC values (*r* = 0.84) were found by Fleishman and Dusek (1971) who tested 90 army enlisted men in a morning and again in an afternoon session. Unfortunately, they do not report the age range of their participants. Even though the test authors declare the PFT to be applicable for grade nine to 16 (Ekstrom et al., 1976), several studies make use of this test in younger populations (e.g., Boakes, 2009; Liben et al., 2013; van der Heyden et al., 2016a). To our knowledge though, no study has evaluated the test–retest reliability of the PFT in younger populations. Harris et al. (2013) state that already children from the age of 5.5 years are able to master the skill of mental paper folding with accuracy increasing with age. They did, however, make use of a paper folding task adapted for younger children. The enhancing impact of age on performance on the PFT is also depicted in our findings, where children achieved lower scores than adolescents and young adults, respectively. These findings are supported by results from van der Heyden et al. (2016b) who applied the PFT in a sample of 217 eight-to-twelve-year-old children and found better results in the older children. Further, in a pilot study with 22 eleven-year-old Dutch children, Bakker (2008) found the PFT to be appropriate for this age group. She translated the instructions, simplified complex sentences, added two sample items to the test and only administered the first half of the test. In line with our study, the instructions were read out loud and a demonstration with a model paper was conducted during instructions.

The MDC_95%_ as a measure to detect clinically significant changes beyond measurement error in repeated measures was rather low ranging between 2.9 and 4.5% in the PFT. These low values across all age groups indicate that the test is a sensitive measure to detect real improvements or deteriorations in performance. We are not aware of comparison values existing in the current literature. When considering practical perspectives, exceedance of these values is indicative of true performance changes. This means, with changes occurring within and above the interval of 2.9 and 4.5% between pre-and post-test, one can be 95% confident that clinically relevant improvements have been detected. When looking at current research, Lowrie et al. (2021) for example found gains of 6.1% in the intervention group compared to the control group in a ten-item digital PFT when testing 641 children and adolescents after twelve lessons of spatial cognitive training. Our findings and the studies mentioned above indicate that the PFT is a reliable instrument to evaluate spatial visualization in older children, adolescents, and young adults and can be utilized to detect intervention changes in these populations.

### 4.2 Mental Rotation Test

The MRT also delivered excellent ICC values for all age groups (0.81–0.84) and small SEM values of 1.9–2.1 points. Similar results were obtained by Kuse (1977) who tested 336 subjects from Hawaiian families (age range: 14–64 years) and reported a strong test–retest reliability of *r* = 0.83 after one year and *r* = 0.70 in an age corrected sample of 456 after one year or more (Vandenberg and Kuse, 1978). While we applied the MRT Version A by Peters et al. (1995b), this test is merely a redrawn version of the stimuli developed by Vandenberg and Kuse (1978). Therefore, the results can be considered comparable. To our knowledge no study evaluated the test–retest reliability in younger children and adolescents. We therefore have to fall back to different test constructs and study designs to discuss our findings.

Hoyek et al. (2012) studied the applicability of the Vandenberg and Kuse MRT compared to a complex two-dimensional MRT in children aged seven to eight years and eleven to twelve years old. They found some evidence that the test might be applicable for the older age group but conclude that both MRT used in their study might be too difficult for the younger children. Similarly, in her pilot study, Bakker (2008) did not find any floor-or ceiling effects when administering the first part of the MRT to eleven-year-old children, concluding its suitability for this age group. According to this author, an adapted time limit of four minutes instead of three minutes which is enabled by the test instructions might however be more suitable for children. For comparability reasons between age groups, we decided to stick with the original time limit of three minutes. Peters himself (personal communication on May 6th, 2021) suggests that the test is suitable from the age of nine onwards. This age is also supported by findings from Titze et al. (2010) who tested fourth graders (aged nine to ten years) on the MRT and found comparable results when comparing the younger with the older children. To make sure, the concept of mental rotation was understood, a two-dimensional test with familiar stimuli was conducted in advance. Additionally Geiser et al. (2008) report considerable improvements in mental rotation performance in terms of items answered correctly and rotation strategy used in 519 children that were tested in grade five (aged ten to eleven years) and then again in grade six (aged eleven to twelve years) suggesting that the ability to mentally rotate three-dimensional stimuli is present by this age.

The MDC_95%_ ranged between 5.2 and 5.9%. To our knowledge, no data for comparison exist in the current literature. Similar values between groups and overall small values are suggestive of a test that detects performance changes with high sensitivity for all three age groups. In terms of practical implication Blüchel et al. (2013) for example found improvements of 6.46% in the intervention-compared to the control group after a two-week motoric intervention to train coordination and orientation in 84 children aged eight to ten years. The current literature in combination with our findings allows for the assumption that the MRT is a suitable measurement tool for the subjects that took part in the present study. When applying the test in younger age groups, adaptations (e.g., extending the time limit, using two-dimensional or more familiar stimuli, etc.) should be considered.

### 4.3 Water Level Task

All age groups obtained excellent test–retest results (0.78–0.88) on the WLT with small SEM values ranging from 1.1–1.3 points. In line with our research, Al-Balushi and Al-Battashi (2013) claim a strong retest reliability (*r* = 0.80) for the WLT when testing 21 female ninth graders from Oman. They do, however, not provide any further information on the testing procedure, the test–retest interval, the testing data, or the subjects. It also needs to be taken into account that significant gender differences have been observed for the WLT with impact of age on the outcomes (for review see Voyer et al., 1995; Pavlovic, 2009). The data by Al-Balushi and Al-Battashi (2013) can therefore merely be considered a rough indication of test–retest reliability of the WLT. Even though this test has been extensively researched over the past decades, we are not aware of any other studies evaluating the test–retest reliability of this measure.

Originally, the WLT was designed to examine the developmental state of spatial concepts in children. According to Piaget the cognitive development of children to successfully handle the WLT should be completed by the age of nine years (Vasta and Liben, 1996). However, while the causes are not completely understood yet, substantial research has detected that significant numbers of adolescents and adults are not able to master this task successfully (e.g., Rebelsky, 1964; Robert and Ohlmann, 1994; Lohaus et al., 1996; Vasta and Liben, 1996). It further needs to be noted that the WLT has only been described anecdotally by Piaget et al. (1956). Therefore, the test stimuli and instructions are usually developed by the researchers and consequently differ across studies lowering comparability. When scoring the test with the criterion method, tolerance levels range between ±4° and ± 10° in the literature (Thomas et al., 1993; Formann, 2003; Merrill et al., 2016). Differences are also found regarding inclination angles, shape of vessels and number of items (Thomas et al., 1993; Pavlovic, 2009; Liben et al., 2013; Merrill et al., 2016). It was therefore essential to confirm the retest reliability of the version used in the present study. This allows to build future research like intervention studies upon a sturdy construct.

MDC_95%_ ranged between 3.0 and 3.6%. Comparable and small values are an indicator of a sensitive measurement tool across age groups. To our knowledge no data for comparison exist in the current literature. From our data, it can be assumed that the WLT is a feasible tool to evaluate spatial perception in the present population, however more research is required to support this conclusion.

### 4.4 Corsi Block Test

Likewise, the CBT delivered excellent test–retest results for the factors span (0.84–0.95) and CS (0.76–0.93) for all age groups. We obtained low SEM values in the range of 0.3–0.4 for the span and 5.0–8.8 for the CS. Again, no age-appropriate base for discussion exists in the current literature. Fisher et al. (2011) performed a digital spatial span task in 64 Scottish children as part of the Cambridge Neuropsychological Test Battery finding a moderate-to-good ICC value of 0.51 and an SEM value of 0.60. While the test composition and conduction were similar to ours, the mean age of the participants was considerably lower (mean age: 6.2 years) and the retest interval significantly longer (three weeks). It is known that visuospatial working memory and CBT performance improve with progressive cognitive development during childhood and adolescence (Pickering, 2001; Farrell Pagulayan et al., 2006) and decline again throughout adulthood (Park and Payer, 2006). The test might thus have been too difficult for the age group used by Fisher et al. (2011). In contrast, Pantoja Cardoso et al. (2023) found similar ICC results to ours (span: 0.72; CS: 0.79) in 35 Brazilian women aged 60–79 years. They report SEM values for span that are comparable to ours (0.50) and that are slightly larger for the CS (18.95). The retest was performed within seven days of the first testing session and test execution was comparable to ours. Dingwall et al. (2017) report slightly lower ICC values (0.64 and 0.65 for agreement and consistency respectively) for the CS of 19 Indigenous Australian adults (mean age: 46.3 years) admitted to a hospital. Again, their testing procedure was comparable to ours and the test–retest interval ranged from one to five days. Interviews with the participants however indicate that language barriers, lack of education, unfamiliarity with computers and lack of concentration due to their current health and social situation might have impacted the performance of this sample. Lower ICC values were detected by White et al. (2019) who report a test–retest reliability of 0.30 for 20 male students aged 18–23 years. The retest was performed in the same testing session and test conduction was similar to the present study. The authors suggest that the poor test–retest reliability might be caused by different levels of sequence difficulty between repeated measures as opposed to identical sequences used in our study.

Oesterlen et al. (2018) tested 387 children (age range: 6–19 years) on a digital CBT. They report an overall strong PCC value of *r* = 0.68. When distinguishing between age groups, the test–retest reliability was *r* = 0.48 for six-to eight-year-olds, *r* = 0.49 for the nine-to twelve-year-olds, and *r* = 0.68 for participants older than twelve years, which is in line with our findings. While the overall execution was similar to the present study, the stimuli were presented as three-dimensional blocks in contrast to our two-dimensional presentation. Moreover, the test was conducted on a tablet compared to a laptop with computer mouse used by us. The same conduction was executed by Williams et al. (2005) who reported a moderate test–retest reliability of *r* = 0.64 when testing 21 subjects aged 12–57 years with an four week test–retest interval. Both, three-dimensional block depictions and indicating the correct order of blocks using a finger are closer to the original conduction of the test were nine cubes are installed on a board and the examiner points a sequence that has to be repeated by the participants by pointing (Corsi, 1972; Kessels et al., 2000).

While a digitalized application of the CBT might help substantially in the accuracy, standardization and administration of the task, the versions used to date still vary considerably in terms of block shape, colors, timing, and devices used (Brunetti et al., 2014; Claessen et al., 2015; Arce and McMullen, 2021). It further needs to be considered that different cognitive processes might be involved when solving a digital as opposed to the original version of the test. Brunetti et al. (2014) and Siddi et al. (2020) found results of a computerized version of the CBT comparable to data of the physical test, Claessen et al. (2015) on the other hand found differences when comparing both tests. More research is needed here to investigate whether the same spatial construct is measured.

We received small MDC_95%_ values between 0.8 and 1.2% for the CBT span and between 13.9 and 24.5% for the CS. We are not aware of any data that can be consulted for comparison. In the context of practical implications, Latino et al. (2021) for example found span improvements of 13.78% for the intervention group over the control group after a twelve week coordinative training in 14-to 15-year-old students. They performed a physical CBT with three trials per block sequence length, two of which had to be repeated correctly. Based on the findings of the present study and the current literature, one can presume that the CBT is a suitable measure for all age groups.

### 4.5 Numbered Cones Run

In an attempt to find an easily reproducible and standardizable measure to evaluate spatial abilities on a larger scale, the NCR delivered excellent test–retest data throughout all age groups (0.86–0.91) and small SEM values (0.3–0.4 s). This measure has been classified as a coordination test for the evaluation of spatial orientation (Hirtz, 1985). As the test has not been used widely in research and has primarily been applied in Eastern European and German speaking countries, reference literature is scarce. Chatzopoulos (2002) reports moderate test–retest reliability (*r* = 0.53) in 43 students aged nine years retested after two weeks. Hirtz et al. (2010) state a test–retest reliability of *r* = 0.78. Both authors, however, do not give any information on how these values were obtained. Considering the insufficient methodological reporting, these findings can thus be only considered a guideline for interpretation of our findings. It needs to be noted, that reference values for interpretation of the test results only exist for children and adolescents aged nine to 15 years (Hirtz et al., 2010). In line with Žamba and Holienka (2014) we adapted the test slightly for feasibility reasons using cones instead of medicine balls. While the different shape and size of the target objects and the placement of the numbers might have influenced the cognitive and motor processes required for this task slightly, we do not expect it to have altered the nature of the test. To date the test has not been described in much detail, therefore tests might vary in terms of placement and mounting of the numbers, showing or calling out of numbers, running sequences or sequences of the target objects (e.g., Chatzopoulos, 2002; Hirtz et al., 2010; Dirksen et al., 2015; Sadowski et al., 2015). Future research is required to confirm our findings and investigate the underlying spatial constructs in more detail.

The NCR appears to be a sensitive tool with MDC_95%_ values ranging from 0.7 to 1.2% between age groups. While this small range of values is indicative of a sensitive measure for all age groups, unfortunately intervention studies using the NCR are scarce or do not report sufficient data to calculate improvements (e.g., Dirksen et al., 2015). The present data implicate that the NCR delivers reproducible data for all age groups.

### 4.6 Limitations and directions for future research

Even though the entire set of tests delivered reproducible test–retest data for all age groups, several limitations of this study need to be noted.

The present study was conducted with healthy children, adolescents, and young adults. Findings are therefore specific to the age groups presented in this study and only applicable for subjects without cognitive impairment. They cannot be generalized to other populations or other spatial ability assessments. Moreover, sample size was rather small. Even though *a priori* power analysis revealed that or sample was sufficient in size, larger studies are needed to confirm our findings and provide a stronger evidence base.

Repetitions of cognitive tests are frequently administered to evaluate intervention effects in, e.g., neuropsychological studies (Beglinger et al., 2005). It needs to be noted, however, that learning-and practice effects in cognitive tests, that is improvements in performance during repeated exposure to the same test stimuli without any intervention, have been discussed in the literature before (e.g., Collie et al., 2003; Beglinger et al., 2005; Scharfen et al., 2018; Fehringer, 2023). This also applies to the tests used in this study. Peters et al. (1995b) found distinct practice effects, when repeating the MRT once per week over the course of four weeks. Practice effects were also reported for the PFT and the CBT (Lohman and Nichols, 1990; Vandierendonck et al., 2004). Possible reasons for practice effects are real skill improvement, skill-related improvements like remembering tasks and answers from the previous test or developing and adapting strategies to solve the tasks, as well as getting accustomed to the test stimuli (Fehringer, 2023). In their recent meta-analysis on effects of spatial learning on mathematics, Hawes et al. (2022) also discuss the possibility that participants could be rearranging their focus and adopt spatial strategies once they have been in contact with even brief spatial ability testing or intervention sessions. With spatial ability testing on three consecutive days, this might also be the case in the present study. Practice effects might differ depending on the cognitive processes required to solve the task. They might further be modified by, e.g., alternating the order of items or using alternative test items in the retest. Having said that, Peters et al. (1995a) observed that engineering students performed significantly better on the MRT (B) (i.e., a version of the MRT that does not differ in terms of procedure and difficulty, but the particular test items vary from the original ones) if they had previous experience with version A of the MRT compared to students that did not have any previous experience with the MRT. For reasons of comparability, we decided to stick with the original versions of the tests. It is further known that longer test–retest intervals tend to reduce practice effects, however practice effects can occur as long as several years after the initial test (Scharfen et al., 2018). In the present study, a brief test–retest interval of 20 min was chosen with a follow-up study on the immediate effects of an acute intervention (i.e., a single motor coordinative exercise session of about 20 min) in mind. Short retest intervals have also been used in previous studies. Participants in a study by Cheng and Mix (2014) performed a 40-min mental rotation training within one week of pretest, followed immediately by the posttest, however average time between pre-and posttest was not provided. Jansen and Richter (2015) evaluated mental rotation performance of children after one hour of creative dance training. Pre-and posttest were performed immediately before and after the intervention. While practice effects occurred in both of these studies, significant changes were only observed in the intervention groups. Bollini et al. (2000) on the other hand do not report any practice effects when evaluating the test–retest reliability of the Dot Test of Visuospatial Working Memory on two consecutive days in participants with schizophrenia and healthy controls.

The high test–retest reliability of all five tests and throughout all age-groups indicates that this approach is suitable to detect true changes. Moreover, memory effects might have been minimized due to the administration of three different cognitive tests with multiple items each in a row. However, when evaluating and interpreting intervention effects in future studies it needs to be considered that practice effects occur. Otherwise, there is a high possibility of overestimating possible intervention effects.

It can further be discussed whether the test–retest interval was adequate to test–retest reliability of the set of tests. Generally speaking, a retest interval should be long enough to rule out memory and practice effects as far as possible as well as fatigue or irritation from the testing procedure. At the same time, it needs to be short enough to obviate improvement or decline in the functions tested due to for example cognitive and physical development (Ritschl et al., 2016; Reynolds et al., 2021). Since for example the results on the WLT can easily be compromised if participants exchange information on their solution approach and the concept of horizontality between test and retest, we decided on a short test–retest interval that would be feasible and easy to control in a regular school-setting. Future research might make use of a longer test–retest interval. Thereby, practice effects could be reduced and good test–retest reliability would strengthen our findings as well as prevent overestimation of intervention effects in future studies.

Moreover, children and adolescents tested in this study happened to be rather close in age. This was due to the availability of consenting children and legal guardians. We intended to recruit children from grade five or six (approximately ten to twelve years) and adolescents from grade eight or nine (approximately 13 to 15 years) since all tests were expected to be suitable for these age groups. For a broader picture it might have been helpful if the mean age of participants was further apart. This issue should be addressed by future research. Moreover, keeping the tremendous cognitive development during childhood and adolescence in mind, large-scale comparisons throughout the developmental process (i.e., including younger children) would be insightful. Here, however, spatial tests would either have to be adapted for younger populations or different measures evaluating the same spatial constructs would have to be selected.

One might also not agree with the decision to utilize the identical spatial ability tests throughout all age groups without taking cognitive developmental differences into consideration. As discussed earlier, it would be a possibility to, e.g., extend time limits on the paper-and-pencil tests for younger populations or decrease the number of items to be completed within a certain time limit. Moreover, it would be possible to use adapted versions of each test for children as they have been used and described in previous literature. In line with previous studies (e.g., Bakker, 2008; Geiser et al., 2008; van der Heyden et al., 2016b; Oesterlen et al., 2018), we decided to maintain the original versions of each test for all three age groups in order to increase comparability between groups. As the results reveal, scores of children tend to be lower than those of adolescents and adults, respectively, as can be expected keeping the developmental process in mind (Table 1). At the same time and keeping the aim of this study in mind, relative and absolute reliability were comparable for all age groups (Table 2). However, when including younger populations in future research, tests might have to be adapted as certain spatial constructs might not be developed sufficiently yet.

Lastly, bearing in mind the definition of large-scale space [i.e., “the space that surrounds the body of the subject standing on the same plane as the spatial layout and that requires the individual to apprehend it from multiple vantage points while moving “(Heil, 2020 based on Weatherford, 1982)], the NCR cannot be considered a large-scale spatial test *per se*, since the entire test space can be viewed from a single position. While it does not provide researchers with the same amount of information about participants’ way finding and navigation behavior as more complex large-scale testing procedures, it still allows to move spatial ability testing to the real world in a standardizable way. It further reveals information on participants real-world spatial orientation behavior. As opposed to virtual reality tests, which are frequently used nowadays to perform reproducible large-scale spatial assessments, the NCR can be performed easily in a school setting with equipment available in every school gym (e.g., Castelli et al., 2008; Wang et al., 2014; Merrill et al., 2016). To test spatial abilities in their entirety, however, a real large-scale test or extrinsic-dynamic test as categorized by Newcombe and Shipley (2015) needs to be included. With VR-solutions becoming more and more available, this might be suitable way to evaluate these spatial skills on a larger scale in the near future. Prerequisite however is that VR-based navigation tests are easily accessible and freely available for all researchers as otherwise no standardized and comparable testing can be conducted (Uttal et al., 2024). One platform that is already providing access to a variety of spatial ability tests is the Spatial Intelligence Learning Center (SILC), which is part of the Northwestern University Research Center.

Different study designs like longitudinal studies might be an additional option to investigate test–retest reliability and spatial abilities throughout the developmental process more thoroughly. Authors like Geiser et al. (2008) already employed this design when evaluating mental rotation performance of children in grade five and then again one year later in grade six. Like this, conclusions can be drawn on the cognitive developmental processes but also information on changes in problem solving strategies might be derived.

As strong sex differences have been reported for, e.g., the MRT but not for other measures by a multitude of studies (e.g., Voyer et al., 1995), taking this topic into account in future research might be insightful in terms of cognitive and spatial ability development. Several factors like brain development, hormones, gender beliefs, exposure to spatial toys and play but also the type of spatial task have been associated with gender differences in spatial abilities (e.g., Kerns and Berenbaum, 1991; Tzuriel and Egozi, 2010; van der Heyden et al., 2016a). It could therefore be of high interest to differentiate between sexes when evaluating test–retest reliability of various spatial abilities in order to see, where sex differences apply. In a next step this could help to develop appropriate interventions to provide children of all sexes with the same opportunities to succeed in STEM-subjects.

## 5 Conclusion

Summarizing it can be said that the excellent relative reliability (high ICC values) and sound absolute reliability (low SEM values) suggest that the entire set of tests investigated in the present study is suitable and delivers reproducible data to evaluate a broad spectrum of spatial abilities in healthy children, adolescents, and young adults. MDC_95%_ values between 0.8 and 24.5% depending on the type of test and the age group represent the amount of change needed between test and retest to detect performance improvements or deteriorations that are relevant to clinical practice.

## Data availability statement

The raw data supporting the conclusions of this article will be made available by the authors, without undue reservation.

## Ethics statement

The studies involving humans were approved by Human Ethics Committee of the University of Duisburg-Essen, Faculty of Educational Sciences. The studies were conducted in accordance with the local legislation and institutional requirements. Written informed consent for participation in this study was provided by the participants’ legal guardians/next of kin.

## Author contributions

CM: Conceptualization, Data curation, Methodology, Writing – original draft, Writing – review & editing. ND: Data curation, Writing – review & editing. AW: Data curation, Writing – review & editing. JW: Data curation, Writing – review & editing. TM: Conceptualization, Data curation, Formal analysis, Methodology, Writing – review & editing.
